# Supplementary material for: An engineered bacterial symbiont allows noninvasive biosensing of the honey bee gut environment
Source: PLoS Biol. 2024 Mar 5;22(3):e3002523. doi: 10.1371/journal.pbio.3002523 (PMC10914260; doi:10.1371/journal.pbio.3002523)
Supplement: S1 Fig — Scatterplot shows linear regression of bacterial concentration values of engineered S. alvi found in matching samples of feces and gut homogenates (i.e., feces and gut were sourced from the same bee). Pearson correlation coefficient R and p-value are provided, for n = 22. The data underlying this Figure can be found in the S1 Data file, sheet “Supplementary Fig 1”. (PDF) [file pbio.3002523.s002.pdf]

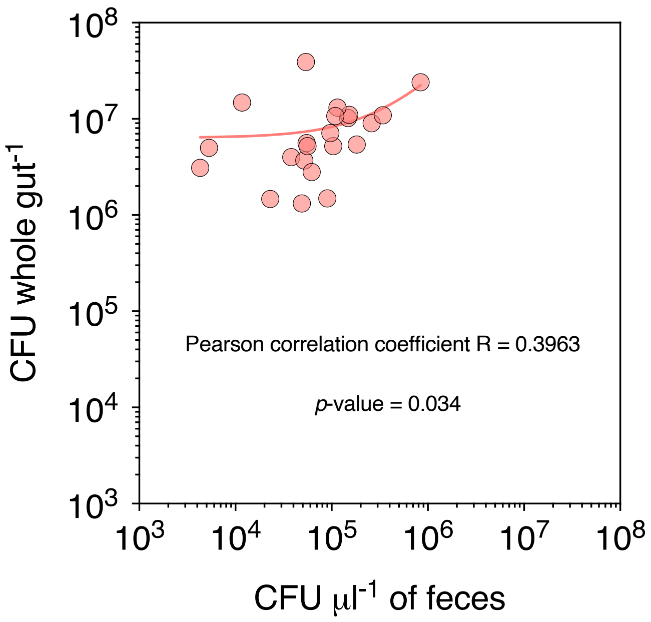

S1 Fig. Bacterial load from feces is a proxy for levels of gut colonization. Scatterplot shows linear regression of bacterial concentration values of engineered *S. alvi* found in matching samples of feces and gut homogenates (*i.e.* feces and gut were sourced from the same bee). Pearson correlation coefficient R and *p*-value are provided, for *n* = 22. The data underlying this Figure can be found in the S1_Data file, sheet "Supplementary Fig1".
