# Supplementary material for: An engineered bacterial symbiont allows noninvasive biosensing of the honey bee gut environment
Source: PLoS Biol. 2024 Mar 5;22(3):e3002523. doi: 10.1371/journal.pbio.3002523 (PMC10914260; doi:10.1371/journal.pbio.3002523)
Supplement: S2 Fig — Broad-host-range replicons were sourced from the pSEVA1213S, pBMTBX-2, pME6012, and pDR401 plasmids (top row). The standard fragments bearing the antibiotic marker and fluorescent protein were obtained from the RSF1010-based vectors pBTK570 [28], pAC08, and pAC09 (left column). In those, RSF1010 was replaced with the different broad-host range replicons, resulting in the 11 new vectors shown. (PDF) [file pbio.3002523.s003.pdf]

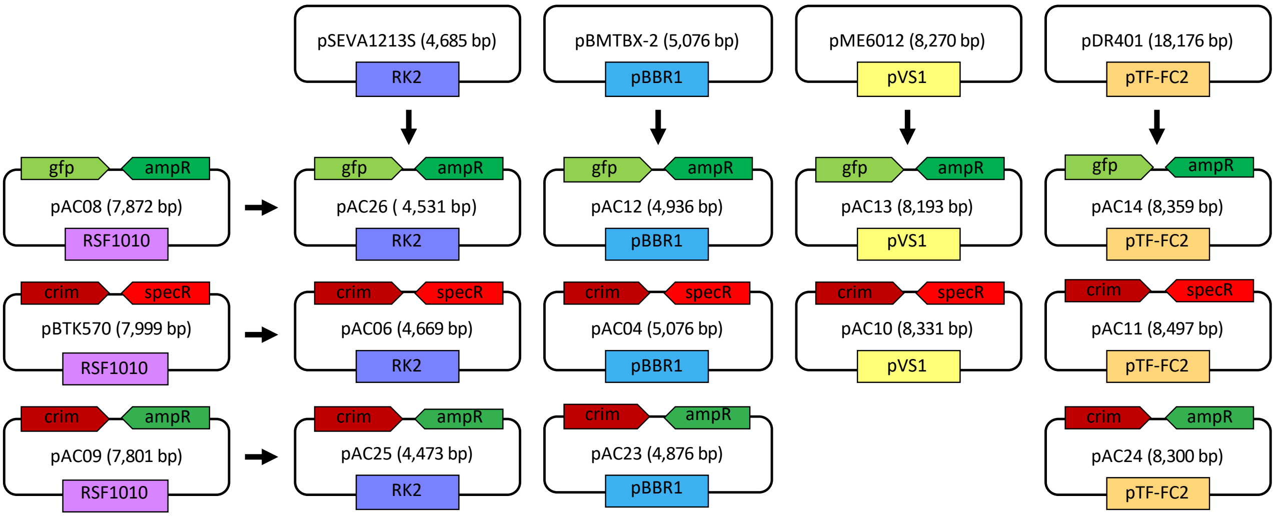

S2 Fig. Plasmid maps of the broad-host range vectors developped in this study*.* Broad-host-range replicons were sourced from the pSEVA1213S, pBMTBX-2, pME6012 and pDR401 plasmids (top row). The standard fragments bearing the antibiotic marker and fluorescent protein were obtained from the RSF1010-based vectors pBTK570 (Leonard *et al.*, 2018), pAC08 and pAC09 (left column). In those, RSF1010 was replaced with the different broad-host range replicons, resulting in the eleven new vectors shown.
