# Supplementary material for: An engineered bacterial symbiont allows noninvasive biosensing of the honey bee gut environment
Source: PLoS Biol. 2024 Mar 5;22(3):e3002523. doi: 10.1371/journal.pbio.3002523 (PMC10914260; doi:10.1371/journal.pbio.3002523)
Supplement: S3 Fig — Primers specificity was confirmed by visualization of amplicons on an agarose gel (left panel) and generation of melting curves (middle panel). Standard curves were generated using serially diluted genomic DNA of (a) S. alvi, (b) B. apis, or (c) miniprep of pBTK570. The data underlying this Figure can be found in the S1 Data file, sheets “Supplementary Fig 3A,” “Supplementary Fig 3B,” and “Supplementary Fig 3C.” (PDF) [file pbio.3002523.s004.pdf]

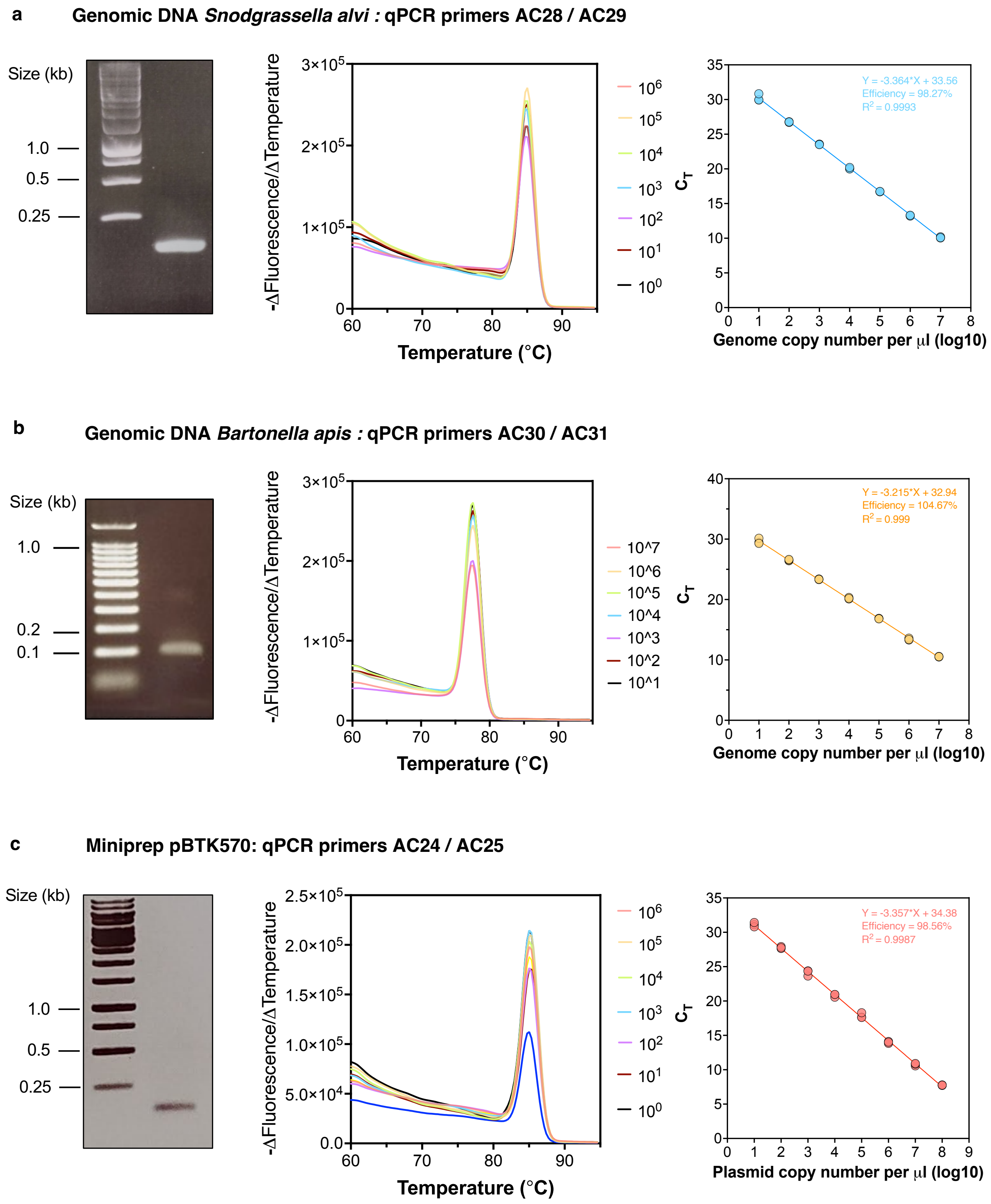

S3 Fig. Validation of qPCR primers and standard curves. Primers specificity was confirmed by visualization of amplicons on an agarose gel (left panel) and generation of melting curves (middle panel). Standard curves were generated using serially diluted genomic DNA of **a** S. alvi, **b** B. apis or **c** miniprep of pBTK570. The data underlying this Figure can be found in the S1_Data file, sheets "Supplementary Fig3a", "Supplementary Fig3b" and "Supplementary Fig3c".
