# Supplementary material for: An engineered bacterial symbiont allows noninvasive biosensing of the honey bee gut environment
Source: PLoS Biol. 2024 Mar 5;22(3):e3002523. doi: 10.1371/journal.pbio.3002523 (PMC10914260; doi:10.1371/journal.pbio.3002523)
Supplement: S4 Fig — (a) Broad-host range plasmids have different copy numbers in B. apis. Box plots show median values of plasmid copy numbers obtained by qPCR from 3 independent experiments with 5 biological replicate each (total n = 15). Median copy number are indicated with the corresponding box plots. (b) The difference in plasmid copy number results in different protein expression levels in B. apis. Graph shows mean of E2-crimson fluorescence ± standard deviations of 5 biological replicates. Each replicate represents the average fluorescence of at least 9,000 cells measured by flow cytometry. Plasmids used for panels a and b in B. apis were pBTK570, pAC06, pAC11, and pAC04, carrying the RSF1010, RK2, pTF-FC2, and pBBR1 origins of replication, respectively. (c) Some replicons are compatible and can be cotransformed in B. apis. Matrix table indicates compatible (green boxes with check mark) and incompatible (red boxes with cross mark) replicons. Vectors were found compatible upon their successful cotransformation by conjugation in B. apis cells. The data underlying this Figure can be found in the S1 Data file, sheets “Supplementary Fig 4A” and “Supplementary Fig 4B.” (PDF) [file pbio.3002523.s005.pdf]

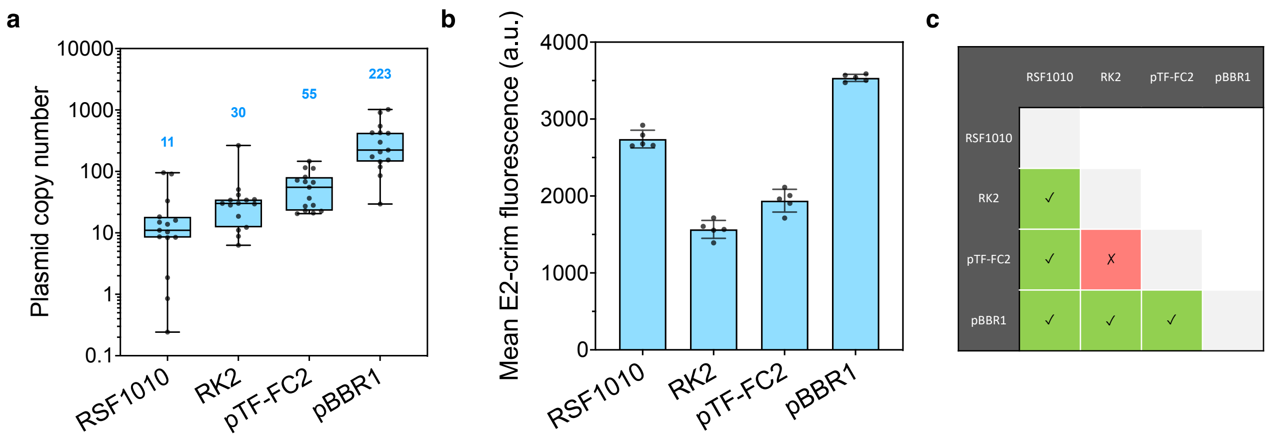

S4 Fig. Characterization of functional broad-host range replicons in the honey bee gut symbiont *B. apis.* a Broad-host range plasmids have different copy numbers in *B. apis.* Box plots show median values of plasmid copy numbers obtained by qPCR from three independent experiments with five biological replicate each (total n=15). Median copy number are indicated with the corresponding box plots. **b** The difference in plasmid copy number results in different protein expression levels in *B. apis.* Graph shows mean of E2-crimson fluorescence $\pm$ standard deviations of five biological replicates. Each replicate represents the average fluorescence of at least 9,000 cells measured by flow cytometry. Plasmids used for panels **a** and **b** in *B. apis* were pBTK570, pAC06, pAC11, and pAC04, carrying the RSF1010, RK2, pTF-FC2 and pBBR1 origins of replication, respectively. **c** Some replicons are compatible and can be co-transformed in *B. apis.* Matrix table indicates compatible (green boxes with check mark) and incompatible (red boxes with cross mark) replicons. Vectors were found compatible upon their successful co-transformation by conjugation in *B. apis* cells. The data underlying this Figure can be found in the S1_Data file, sheets "Supplementary Fig4a" and "Supplementary Fig4b".
