# Supplementary material for: An engineered bacterial symbiont allows noninvasive biosensing of the honey bee gut environment
Source: PLoS Biol. 2024 Mar 5;22(3):e3002523. doi: 10.1371/journal.pbio.3002523 (PMC10914260; doi:10.1371/journal.pbio.3002523)
Supplement: S5 Fig — (a) Graph shows mean ± standard deviation percentage of bacterial population GFP positive. Three biological replicates were tested for each construct. Each replicate value is based on the average fluorescence of at least 9,000 S. alvi cells measured by flow cytometry, which were grown in 3 ml of TSB with (+) or without (−) IPTG for 3 days. As a reference, S. alvi bearing the pAC08 plasmid constitutively expressing GFP and S. alvi carrying our pAC17V5 dual-vector system were also analyzed. (b) The CP25 interregion gets deleted from pBTK552. Plasmid map of the pBTK552 vector (top panel) with a representative Sanger sequencing of a frequent deletion obtained in the unstable region (bottom panel) is shown. Dotted lines indicate the position of the deletion. The data underlying this Figure can be found in the S1 Data file, sheet “Supplementary Fig 5A.” (PDF) [file pbio.3002523.s006.pdf]

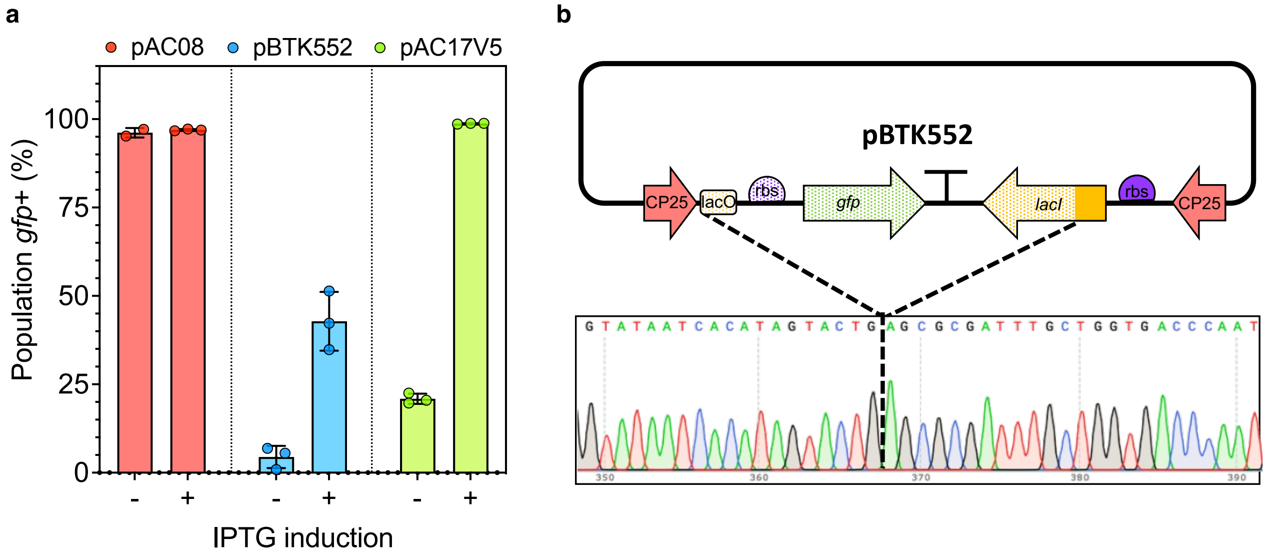

S5 Fig. The plasmid pBTK552 (Leonard *et al*., 2018)[28] is genetically unstable in our experimental conditions. a Graph shows mean ± standard deviation percentage of bacterial population GFP positive. Three biological replicates were tested for each construct. Each replicate value is based on the average fluorescence of at least 9,000 *S. alvi* cells measured by flow cytometry, which were grown in 3 ml of TSB with (+) or without (-) IPTG for 3 days. As a reference, *S. alvi* bearing the pAC08 plasmid constitutively expressing GFP and *S. alvi* carrying our pAC17V5 dual-vector system were also analyzed. **b** The CP25 inter region gets deleted from pBTK552. Plasmid map of the pBTK552 vector (top panel) with a representative sanger sequencing of a frequent deletion obtained in the unstable region (bottom panel) are shown. Dotted lines indicate the position of the deletion. The data underlying this Figure can be found in the S1_Data file, sheet "Supplementary Fig5a".
