# Supplementary material for: An engineered bacterial symbiont allows noninvasive biosensing of the honey bee gut environment
Source: PLoS Biol. 2024 Mar 5;22(3):e3002523. doi: 10.1371/journal.pbio.3002523 (PMC10914260; doi:10.1371/journal.pbio.3002523)
Supplement: S6 Fig — (a) Maps of the IPTG-inducible plasmids built in this study. (b) S. alvi cells engineered with our inducible plasmids respond to IPTG exposure in vitro. Graph shows box plots representing median value of GFP fluorescence of 5 biological replicates for each construct tested. Each replicate value is based on the average fluorescence of at least 9,000 S. alvi cells measured by flow cytometry, which were grown in liquid with (+) or without (−) IPTG. As a reference, wild-type S. alvi, S. alvi bearing the pAC08 plasmid constitutively expressing GFP, and S. alvi carrying the previously built pBTK552 vector [28] were also analyzed. Fold-changes of average fluorescence between uninduced and induced cells are indicated. The data underlying this Figure can be found in the S1 Data file, sheet “Supplementary Fig 6B.” (PDF) [file pbio.3002523.s007.pdf]

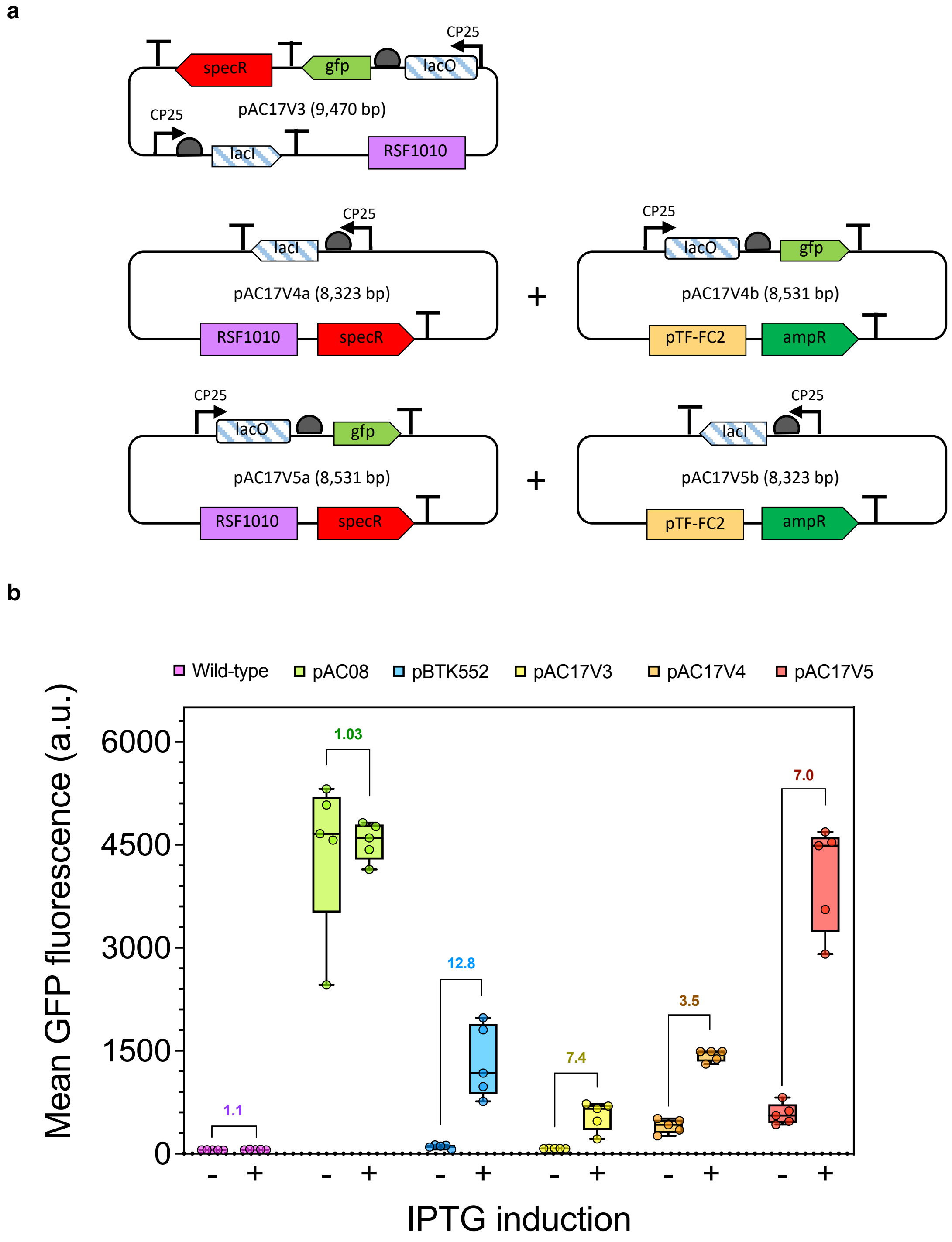

S6 Fig. Testing of different IPTG-inducible constructs in *S. alvi*. a Maps of the IPTG-inducible plasmids built in this study. **b** *S. alvi* cells engineered with our inducible plasmids respond to IPTG exposure *in vitro*. Graph shows box plots representing median value of GFP fluorescence of five biological replicates for each construct tested. Each replicate value is based on the average fluorescence of at least 9,000 *S. alvi* cells measured by flow cytometry, which were grown in liquid with (+) or without (-) IPTG. As a reference, wild-type *S. alvi*, *S. alvi* bearing the pAC08 plasmid constitutively expressing GFP and *S. alvi* carrying the previously built pBTK552 vector (Leonard *et al*., 2018)[28] were also analyzed. Fold-changes of average fluorescence between uninduced and induced cells are indicated. The data underlying this Figure can be found in the S1_Data file, sheet "Supplementary Fig6b".
