# Supplementary material for: An engineered bacterial symbiont allows noninvasive biosensing of the honey bee gut environment
Source: PLoS Biol. 2024 Mar 5;22(3):e3002523. doi: 10.1371/journal.pbio.3002523 (PMC10914260; doi:10.1371/journal.pbio.3002523)
Supplement: S7 Fig — Snodgrassella alvi ESL0693, Gilliamella apicola wkB7, and Gilliamella apis ESL0169 cells engineered with the inducible plasmid pAC17V3 respond to IPTG exposure in vitro. Graphs show box plots representing median value of GFP fluorescence of 5 biological replicates for each condition. Each replicate value is based on the fluorescence of cells grown as bacterial lawns onto solid media (TSA) supplemented with (+) or without (−) 1 mM IPTG. Fold-changes of average fluorescence between uninduced and induced cells are indicated. Fluorescence was determined from images taken by EPI fluorescence with a Fusion FX (Vilber Lourmat) apparatus (16 aperture, F-740 filter) and identical exposure times between conditions. Analysis was performed with the software Fiji (ImageJ2, version 2.9.0). The intensity measurement used was the mean intensity per unit area of bacterial lawn with the cell and media autofluorescence subtracted. Autofluorescence values were obtained for each strain based on the intensity measured from bacterial lawns of the corresponding wild-type cells (i.e., not bearing the pAC17V3 plasmid). The data underlying this Figure can be found in the S1 Data file, sheet “Supplementary Fig 7.” (PDF) [file pbio.3002523.s008.pdf]

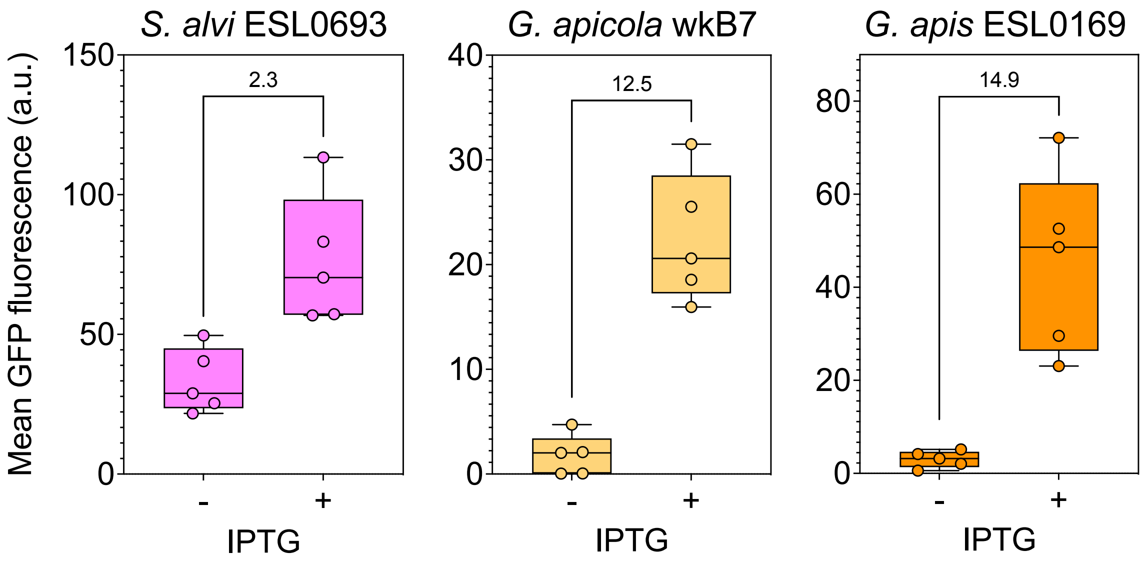

S7 Fig. The IPTG-inducible construct pAC17V3 allows honey bee and stingless bee gut symbionts to sense and respond to IPTG. *Snodgrassella alvi* ESL0693, *Gilliamella apicola* wkB7 and *Gilliamella apis* ESL0169 cells engineered with the inducible plasmid pAC17V3 respond to IPTG exposure *in vitro*. Graphs show box plots representing median value of GFP fluorescence of five biological replicates for each condition. Each replicate value is based on the fluorescence of cells grown as bacterial lawns onto solid media (TSA) supplemented with (+) or without (-) 1 mM IPTG. Fold-changes of average fluorescence between uninduced and induced cells are indicated. Fluorescence was determined from images taken by EPI fluorescence with a Fusion FX (Vilber Lourmat) apparatus (16 aperture, F-740 filter) and identical exposure times between conditions. Analysis was performed with the software Fiji (ImageJ2, version 2.9.0). The intensity measurement used was the mean intensity per unit area of bacterial lawn with the cell and media autofluorescence subtracted. Autofluorescence values were obtained for each strain based on the intensity measured from bacterial lawns of the corresponding wild-type cells (*i.e.* not bearing the pAC17V3 plasmid). Cells fluorescence was measured from bacterial lawn rather than using flow cytometry, due to the growth of these bacterial strains as dense aggregates that results in non-reliable flow cytometry data. The data underlying this Figure can be found in the S1_Data file, sheet “Supplementary Fig7”.
