# Supplementary material for: An engineered bacterial symbiont allows noninvasive biosensing of the honey bee gut environment
Source: PLoS Biol. 2024 Mar 5;22(3):e3002523. doi: 10.1371/journal.pbio.3002523 (PMC10914260; doi:10.1371/journal.pbio.3002523)
Supplement: S8 Fig — Graph shows box plots representing median value of DAPI fluorescence of S. alvi biofilms imaged from the gut of bees fed sugar water supplemented with either 0, 0.1, or 1 mM IPTG. Five bees were analyzed for each condition, and fluorescence values were averaged from 3 distinct sections of each gut. One-way ANOVA test, not significant (ns) with q-value > 0.5. The data underlying this Figure can be found in the S1 Data file, sheet “Supplementary Fig 8.” (PDF) [file pbio.3002523.s009.pdf]

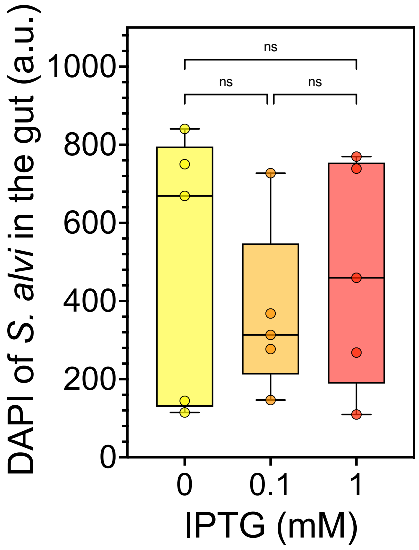

S8 Fig. DAPI measurements from gut tissues. Graph shows box plots representing median value of DAPI fluorescence of *S. alvi* biofilms imaged from the gut of bees fed sugar water supplemented with either 0, 0.1 or 1 mM IPTG. Five bees were analyzed for each condition and fluorescence values were averaged from three distinct sections of each gut. One-way ANOVA test, not significant (ns) with *q*-value > 0.5. The data underlying this Figure can be found in the S1_Data file, sheet "Supplementary Fig8".
