# Supplementary material for: An engineered bacterial symbiont allows noninvasive biosensing of the honey bee gut environment
Source: PLoS Biol. 2024 Mar 5;22(3):e3002523. doi: 10.1371/journal.pbio.3002523 (PMC10914260; doi:10.1371/journal.pbio.3002523)
Supplement: S9 Fig — Graphs show pseudocolor plots used for gating of S. alvi cells. Quadrant limits were determined based on the measured fluorescence of reference cells bearing (a) the empty backbone pAC07 (no fluorescence), (b) pAC08 (GFP alone), or (c) pBTK570 (E2-crimson alone). (PDF) [file pbio.3002523.s010.pdf]

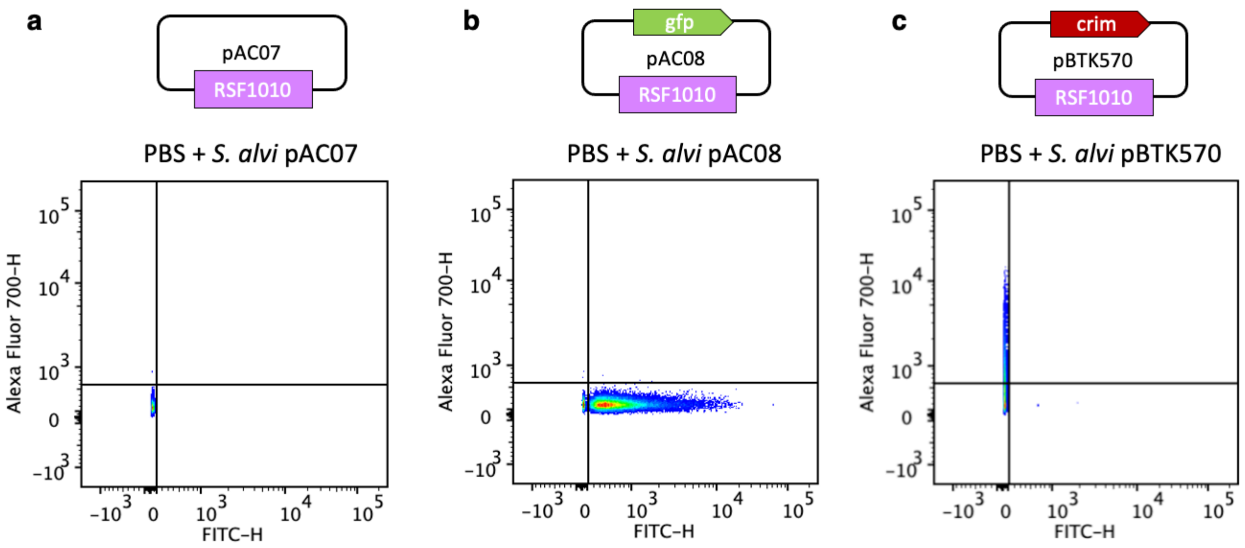

S9 Fig. Cell gating for flow cytometry analysis. Graphs show pseudocolor plots used for gating of *S. alvi* cells. Quadrant limits were determined based on the measured fluorescence of reference cells bearing **a** the empty backbone pAC07 (no fluorescence), **b** pAC08 (GFP alone) or **c** pBTK570 (E2-crimson alone)**.**
