# Supplementary material for: An engineered bacterial symbiont allows noninvasive biosensing of the honey bee gut environment
Source: PLoS Biol. 2024 Mar 5;22(3):e3002523. doi: 10.1371/journal.pbio.3002523 (PMC10914260; doi:10.1371/journal.pbio.3002523)
Supplement: S1 Table — (PDF) [file pbio.3002523.s011.pdf]

Supplementary Table 1. **Plasmids used in this study.**

Plasmid	Description	Reference or source
pBTK503	pBTK plasmid with constitutive GFP, RSF1010 replicon	Addgene No. 110616 [1]
pBTK570	pBTK plasmid with constitutive E2-crimson, RSF1010 replicon	Addgene No. 110615 [1]
pBTK552	pBTK plasmid with IPTG-inducible GFP, RSF1010 replicon	Addgene No. 110618 [1]
pBMTBX-2	Template plasmid carrying the pBBR1 replicon	Addgene No. 26073 [2]
pME6012	Template plasmid carrying the pVS1 replicon	Heeb *et al*., 2000 [3]
pDR401	Template plasmid carrying the pTF-FC2 replicon	Rawlings *et al.*, 1984 [4]
pSEVA1213S	Template plasmid carrying the RK2 replicon	Addgene No. 122095 [5]
pAC08	Constitutive GFP, ampR, RSF1010 replicon	This study
pAC09	Constitutive E2-crimson, ampR, RSF1010 replicon	This study
pAC26	Constitutive GFP, ampR, RK2 replicon	This study
pAC06	Constitutive E2-crimson, specR, RK2 replicon	This study
pAC25	Constitutive E2-crimson, ampR, RK2 replicon	This study
pAC12	Constitutive GFP, ampR, pBBR1 replicon	This study
pAC04	Constitutive E2-crimson, specR, pBBR1 replicon	This study
pAC23	Constitutive E2-crimson, ampR, pBBR1 replicon	This study
pAC13	Constitutive GFP, ampR, pVS1 replicon	This study
pAC10	Constitutive E2-crimson, specR, pVS1 replicon	This study
pAC14	Constitutive GFP, ampR, pTFC-FC2 replicon	This study
pAC11	Constitutive E2-crimson, specR, pTFC-FC2 replicon	This study
pAC24	Constitutive E2-crimson, ampR, pTFC-FC2 replicon	This study
pAC17V5a	IPTG-inducible two-plasmid system bearing CP25 > lacO-GFP, specR, RSF1010 replicon (1/2)	This study
pAC17V5b	IPTG-inducible two-plasmid system bearing CP25 > lacI, ampR, pTF-FC2 replicon (2/2)	This study

References

1. Leonard SP, Perutka J, Powell JE, Geng P, Richhart DD, Byrom M, et al. **Genetic Engineering of Bee Gut Microbiome Bacteria with a Toolkit for Modular Assembly of Broad-Host-Range Plasmids**. *ACS Synth Biol*. 2018;7(5):1279–90.
2. Prior JE, Lynch MD, Gill RT. **Broad-host-range vectors for protein expression across gram negative hosts**. *Biotechnol Bioeng*. 2010;106(2):326–32.
3. Heeb S, Itoh Y, Nishijyo T, Schnider U, Keel C, Wade J, et al. **Small, stable shuffle vectors based on the minimal pVS1 replicon for use in gram-negative, plant-associated bacteria**. *Mol Plant-Microbe Interact*. 2000;13(2):232–7.
4. Rawlings DE, Pretorius I, Woods DR. **Expression of a Thiobacillus ferrooxidans origin of replication in *Escherichia coli***. *J Bacteriol*. 1984;158(2):737–8.
5. Wirth NT, Kozaeva E, Nikel PI. **Accelerated genome engineering of *Pseudomonas putida* by I-SceI—mediated recombination and CRISPR-Cas9 counterselection.** *Microb Biotechnol.* 2020;13(1):233–49.
