# Supplementary material for: An engineered bacterial symbiont allows noninvasive biosensing of the honey bee gut environment
Source: PLoS Biol. 2024 Mar 5;22(3):e3002523. doi: 10.1371/journal.pbio.3002523 (PMC10914260; doi:10.1371/journal.pbio.3002523)
Supplement: S2 Table — Uppercase letters indicate priming sequence, and lowercase nucleotides show homology regions for Gibson assembly as primer overhangs. (PDF) [file pbio.3002523.s012.pdf]

Supplementary Table 2. Primers used in this study. Uppercase letters indicate priming sequence and lowercase nucleotides show homology regions for Gibson assembly as primer overhangs.

Primers	Sequences (5' – 3')	Description	Resulting plasmid
AC09	ATCTGAATCATGCGCGGATG	Linearize pAC08/pAC09/pBTK570 without replicon	N/A
AC10	CACGTTAAGGGATTTTGGTCATG		
AC16	gaccaaaatcccttaacgtgCCAAAGGGTTCGTGTAGACT	Amplify RK2 from pSEVA1213S	pAC06 / pAC26 / pAC25
AC17	catccgcgcatgattcagatGCGCGCCGTAGAAAAGATC		
AC11	gaccaaaatcccttaacgtgACGCCCGGTAGTGATCTTAT	Amplify pBBR1 from pBMTBX-2	pAC12 / pAC04 / pAC23
AC12	catccgcgcatgattcagatGACGAGCCTCAGACTCCAG		
AC48	catccgcgcatgattcagatCTGGTTTCGGAGTTGACAGC	Amplify pTF-FC2 (fragment 1/2) from pDR401	pAC14 / pAC11 / pAC24 / pAC17V5b
AC49	CGTCTTTGATAAGCCGCTCC		
AC50	GGAGCGGCTTATCAAAGACG	Amplify pTF-FC2 (fragment 2/2) from pDR401	
AC51	gaccaaaatcccttaacgtgCTCAAGGCAGCCAGAACATC		
AC46	GAAACCGCCATCAGTACCAG	Amplify pVS1 (fragment 1/2) from pME6012	pAC13 / pAC10
AC44	gaccaaaatcccttaacgtgTTCCTGGCGTTTTCTTGTCG		
AC45	catccgcgcatgattcagatGTAGACAACATCCCCTCCCC	Amplify pVS1 (fragment 2/2) from pME6012	
AC47	CTGGTACTGATGGCGGTTTC		
AC36	CGAGCGGCCGCGATTATC	Linearize pBTK503 (fragment 1/2 for pAC08 and 1/3 for pAC09)	pAC08 / pAC09
AC39	GTCAAGCGCTTCGGTCATG		
AC37	ttgataatcgcggccgctcgCCACTGACGAGCAGATTTCCAG	Linearize pBTK503 (fragment 2/2) for pAC08	pAC08
AC38	CATGACCGAAGCGCTTGAC		
AC41	ttgataatcgcggccgctcgAGTATCCGGTTCCCATCTGC	Linearize pBTK570 (fragment 2/3)	pAC09
AC42	ctttggcagtttattcttgacatgtagtgagggggctggtataatcacatagtactgttATACAGAAACAGAGGAGATATTACATATGG		
AC43	ttgataatcgcggccgctcgCCACTGACGAGCAGATTTCCAG	Linearize pBTK570 (fragment 3/3), to use with AC38	
AC_59	GGTAGCCATTGATTGCCTCC	Linearize pBTK552 (fragment 1/2)	pAC17V5a
AC_114	gtagtcggcaaataagcggcGTGAACACTCTCCCGGTTGT		
AC_60	GGAGGCAATCAATGGCTACC	Linearize pBTK552 (fragment 2/2)	
AC_70	GCCGCTTATTTGCCGACTAC		
AC_115	gatgacacgaactcacgacgCGCTCGGCTTTGGCAGTTTATTC	Amplify *lacI* from pBTK552, to clone with pTF-FC2	pAC17V5b
AC_116	ttgataatcgcggccgctcgAAACAAAAGGCCCAGTCTTCC		

Primers	Sequences (5' – 3')	Description	Reference or source
AC30	gtgggaatctacctatttctacg	qPCR primers binding the 16S region of *B. apis*	Kesnerova *et al.*, 2017[1]
AC31	aacgcgggctcatctatctc		
AC28	cttagagataggagagtgcctt	qPCR primers binding the 16S region of *S. alvi*	Kesnerova *et al.*, 2017[1]
AC29	aacttaatgatggcaactaatgacaa		
AC24	ctccaaggcgtacatcaagc	qPCR primers binding the E2-crimson	This study
AC25	gtcctcgaagttcatcacgc		

References

1. Kešnerová L, Mars RAT, Ellegaard KM, Troilo M, Sauer U, Engel P. **Disentangling metabolic functions of bacteria in the honey bee gut**. *PLOS Biol*. 2017;15(12):1–28.
