# Supplementary material for: An engineered bacterial symbiont allows noninvasive biosensing of the honey bee gut environment
Source: PLoS Biol. 2024 Mar 5;22(3):e3002523. doi: 10.1371/journal.pbio.3002523 (PMC10914260; doi:10.1371/journal.pbio.3002523)
Supplement: S1 Text — (PDF) [file pbio.3002523.s013.pdf]

S1 Text. Fiji macro.

```
dir=getDirectory("Choose Source Dir");
File.makeDirectory(dir+"results/");
File.makeDirectory(dir+"results/GFPmax/");
list=getFileList(dir);
Array.show(list);

function makeCellmask(id){
				print("Creating cell mask from GFP channel for " + id);
				open(dir + id);
				selectWindow(id+" - C=1");
				close();
				selectWindow(id+" - C=0");
				run("Z Project...", "projection=[Max Intensity]");

				run("8-bit");
				run("Duplicate...", "title=["+id+"- Cell mask]");
				run("Duplicate...", "title=["+id+"- Cell peaks]");

				selectWindow(id+"- Cell mask");
				setAutoThreshold("Triangle dark");
				setThreshold(19, 255);
				run("Convert to Mask");

				selectWindow(id+"- Cell peaks");
				run("Duplicate...", "title=["+id+"- Cell signal]");
				saveAs("Tiff", dir+"results/"+id+"- Cell signal");
				wait(500);
				run("Close");

				selectWindow(id+"- Cell peaks");
				run("Median...", "radius=1");
				run("Gaussian Blur...", "sigma=0.20 scaled");
				run("Find Maxima...", "prominence=25 exclude output=[Segmented
Particles]");
				run("Invert");
				wait(500);
				imageCalculator("Subtract create", id+"- Cell mask", id+"- Cell peaks
Segmented");
				saveAs("Tiff", dir+"results/"+id+"- Cell mask");
				wait(500);
				run("Close");
				close(id+"- Cell mask.tif");
				close("\\Others");
				while (nImages>0) {
	selectImage(nImages);
	close();
   }
}

function separatepercell(id){
				print("Creating ROIs for each cell in " + id);
```

```
			open(dir +"results/"+ id +"- Cell mask.tif");
			run("Analyze Particles...", "size=0.8-Infinity circularity=0-1.00 display exclude
clear summarize add");
			run("Clear Results");
			selectWindow("Summary");
			run("Close");
			cells = roiManager("count");
			print("There are " +cells+" cells.");
			wait(1);
			for (c=0; c<cells; c++){
				roiManager("Select", c);
				roiManager("rename", roiManager("index"));
				wait(1);
			}
			roiManager("save", dir+"results/"+id+".zip");
			selectWindow("ROI Manager");
			run("Close");


			for (c=0; c<cells; c++){
				roiManager("reset");
				roiManager("open", dir+"results/"+id+".zip");
				open(dir +"results/"+ id +"- Cell signal.tif");
				wait(50);
				roiManager("Select", c);
				run("Crop");
				run("Measure");
				}
			selectWindow("ROI Manager");
			run("Close");
			selectWindow("Results");
			saveAs("Text", dir+"results/GFPmax/"+id+"_GFPtable.txt");
			wait(50);
			run("Close");
			while (nImages>0) {
				selectImage(nImages);
				close();
			}
			wait(100);
}


			for (l=0; l<list.length; l++){
			print("Starting :"+list[l]);
			if (endsWith(list[l], "/")){
				print("This is a directory");
				}
			else{
			if (endsWith(list[l], ".czi")){
				run("Clear Results");
				id = list[l];
				makeCellmask(id);
				separatepercell(id);
```

```
                        }
                }
                wait(1);
                close("\\Others");
                while (nImages>0) {
                selectImage(nImages);
                close();
        }
}
```
