# Supplementary material for: An engineered bacterial symbiont allows noninvasive biosensing of the honey bee gut environment
Source: PLoS Biol. 2024 Mar 5;22(3):e3002523. doi: 10.1371/journal.pbio.3002523 (PMC10914260; doi:10.1371/journal.pbio.3002523)
Supplement: Attachment — Submitted filename: AChhun_Rebuttal_PLoS-Biology.pdf [file pbio.3002523.s014.pdf]

Response to Reviewers:

Engineering a symbiont as a biosensor for the honey bee gut environment.

Dear Dr Schaerli,

Thank you for your patience while your manuscript "Engineering a symbiont as a biosensor for the honey bee gut environment" was peer-reviewed at PLOS Biology. Please accept my sincere apologies for the delays that you have experienced during the peer review process. Your manuscript has now been evaluated by the PLOS Biology editors, an Academic Editor with relevant expertise, and by three independent reviewers.
In light of the reviews, which you will find at the end of this email, we would like to invite you to revise the work to thoroughly address the reviewers' reports.

As you will see below, Reviewers #1 and #3 are generally positive about the method and think that the IPTG biosensor will be useful for the field. Reviewer #2 raises concerns with the overall strength of the technical advance and notes that the impact of the work could be enhanced by further generalizing the method, such as demonstrating the utility of the biosensor in another bee-related bacterium or by developing another biosensor that can sense natural compounds. Reviewer #3 agreed with this comment during reviewer cross-commenting that we offer at the journal. After discussions with the Academic Editor, we feel that the development of a new biosensor is outside the scope for a revision, but we agree that incorporating data showing the application of the IPTG biosensor to other bee-related bacteria would strengthen the manuscript.
Given the extent of revision needed, we cannot make a decision about publication until we have seen the revised manuscript and your response to the reviewers' comments. Your revised manuscript is likely to be sent for further evaluation by all or a subset of the reviewers.

We expect to receive your revised manuscript within 3 months. Please email us (plosbiology@plos.org) if you have any questions or concerns, or would like to request an extension.

Please submit the following files along with your revised manuscript:

1. A 'Response to Reviewers' file - this should detail your responses to the editorial requests, present a point-by-point response to all of the reviewers' comments, and indicate the changes made to the manuscript. *NOTE: In your point-by-point response to the reviewers, please provide the full context of each review. Do not selectively quote paragraphs or sentences to reply to. The entire set of reviewer comments should be present in full and each specific point should be responded to individually, point by point.

You should also cite any additional relevant literature that has been published since the original submission and mention any additional citations in your response.
2. In addition to a clean copy of the manuscript, please also upload a 'track-changes' version of your manuscript that specifies the edits made. This should be uploaded as a "Revised Article with Changes Highlighted" file type.

We thank the reviewers and editors for their valuable feedback. Please find below our detailed response that addresses all comments/suggestions. Most importantly, we included now new data showing that our IPTG biosensor not only functions in *S. alvi*, but also works in γ-Proteobacteria from the honey bee gut microbiota, namely *Gilliamella apicola* wkB7 and *Gilliamella apis* ESL0169, as well as in another strain of *S. alvi* isolated from stingless bees (see new Supplementary Fig. 7, and our detailed response to Reviewer 2).

Concerning additional relevant literature, we now mention recent work by Lariviere and colleagues who established a new method based on electroporation and homologous recombination to perform one-step genomic deletions and integrations in *S. alvi* (Lariviere et al., 2023), which we included (see lines 68-70) to provide an up-to-date introduction on the state of the art. We also added in our discussion a mention to our own most recent pre-print in which we characterized the fecal microbiota of the honey bee (Cabirol et al., 2023), thus confirming that *S. alvi* (and other bacteria settling in the proximal gut) accumulates in the rectum and can be found in the feces (see lines 384-386). Lastly, we added a News & Views article highlighting the challenges of engineering environmental undomesticated bacteria (see line 424; Libby & Silver, 2019).

<u>Reviewer #1:</u> The manuscript by Chhun and colleagues describes a new genetic toolkit for the honey-bee, allowing for approaches to study host-gut microbiota interactions. The authors also engineered the native bee gut bacterium Snodgrassella alvi as a biosensor for IPTG and showed that fluorescence readout can be measured in the gut tissues or non-invasively in the faeces.

Overall, I think that this is an exciting and environmentally important model system that is currently underdeveloped. Tools such as these could open up a wide range of studies in terms of biological understanding and provide novel modalities for manipulation. I have some suggestions/comments for the improvement of the manuscript.

Authors: We thank reviewer 1 for their constructive suggestions/comments. We amended the manuscript to reflect the reviewer' suggestions, when possible.

- Line 32: There are also electronic auto sampling devices that can be used which may be termed non-invasive. I think these should be mentioned.

  Authors: Following the reviewer' suggestion, we have modified our introduction to now mention recent relevant literature on ingestible sampling devices as well as other non-invasive methods (see **line 29-32: "***Gaining insight into host-microbiota interactions is, however, inherently arduous because the gut environment is not readily*

*accessible as its content can only be queried by dissection of the gut tissue, invasive sampling methods, **ingestible sampling devices** or indirectly by sampling fecal matter **or analyzing the volatilome of the host (2–5)***”).

- Figure 1d: Although the Kaplan-Meier test gives an insignificant p value from the curve it looks like there is an impact on fitness and perhaps that further sampling may be detrimental. Perhaps this could be commented on in the discussion, saying further study is warranted.

  Authors: In response to a comment made in a previous peer-review at another journal, we already added the point raised by the reviewer in our discussion, see **lines 397-403**: “*Our experiments also suggest that repeated feces collection does not significantly impact the fitness of honey bees, as no statistically significant difference in survival between non-treated and treated bees was measured. However, we observed a trend in which treated individuals might die earlier than non-treated bees, most likely because of moderate stress caused by handling during feces collection. Therefore, further studies investigating the precise impact of feces collection on bee health might be warranted.*”

- Line 201: You mention plasmid maintenance systems here and in the discussion. You know there is one on pTF-FC2. Are there others on the plasmids you are using? Could you consider adding maintenance systems to the other plasmids?

  Authors: The pTF-FC2 replicon is the only one from our plasmid collection to bear a maintenance system (*i.e.* toxin/antitoxin system). We discussed adding this maintenance system to other plasmids, see **lines 369-373**: “*Interestingly, the low-copy number pTF-FC2 vector has a toxin/antitoxin system naturally encoded within the replicon itself that surely contributes to this lasting plasmid maintenance(46). Based on three genes of small sizes (~700 bp), the system could easily be ported to other plasmids and could therefore potentially modulate their propagation and maintenance*”.

Reviewer #2 (Tae Seok Moon, signs review): In this work, the authors claim that they could measure fluorescence in the gut tissues or in the feces of honey bees by reprogramming a native bee gut bacterium as a biosensor for IPTG. It is an interesting paper with unusual environments and topics of interest (e.g., bee feces and symbionts). Conceptually, this work would be novel with interesting angles of research. Technically, however, the work is a bit simple as is; too simple to make a big impact or significant advances in synthetic biology. As is, the work would attract a limited number of readers. The following comments are given to make it stronger.

Authors: We thank the reviewer for their constructive feedback on our manuscript. We addressed each point raised below. As a general response, however, we would like to clarify that our goal was to develop new tools and methods to use a synthetic biology approach for the development of biosensors for the gut environment of honey bees. We hope readers will be interested not only by the synthetic biology angle but especially by the advance our study represents for the field of the honey bee gut microbiota. We understand that reading our work through the lens of the synthetic biology field and thinking of typical chassis like *Escherichia coli*, one might find it very simple. However, we think one should consider our work is based on poorly genetically tractable bacteria, where presumably "simple" things, such as creating a stable plasmid coding for a fluorescent reporter, can be very challenging.

1) There are many practical biosensors that have been developed, mostly for applications in the mouse gut microbiome. Although this work focuses on honey bees' microbiota, it would be great for the authors to expand their discussion on biosensors developed for microbiome engineering and applications.

   Authors: We have expanded our discussion on biosensors developed for microbiome engineering (see **lines 309-319**: "*A striking advantage of bacterial biosensors is that they allow to non-invasively and non-destructively examine the gut content and its associated bacterial communities. In less than a decade, the development of technologies to reprogram human commensals like E. coli as biosensors has seen exciting progress, starting from the use of transcriptional regulator-based genetic circuits to sense simple chemicals(9), to the application of CRISPR to make bacteria record in their genomes transcriptional changes occurring as they pass through the guts(43).* ***For instance, it was shown that the human commensal Bacteroides thetaiotaomicron could be genetically reprogrammed to sense and respond to arabinogalactan supplemented in the food of colonized mice(10). Similarly, the probiotic E. coli Nissle 1917 was engineered to simultaneously sense thiosulfate and nitrate in the gastrointestinal tract of mice as biomarkers for gut inflammation(11).***")

2) The IPTG sensor developed here might be novel in the specific species, but it is not practically interesting or useful, given the two-decade-old history of synthetic biology, generating multiple practical sensors. This work would have a bigger impact if the authors demonstrate the sensor's generalizability (e.g., demonstrating the functional IPTG sensor in another bee-related bacterium) or a more useful sensor for practical applications. Alternatively, they could develop another sensor in the same bacterium

(e.g., sensors for anhydrotetracycline [aTc] although aTc is not interesting or useful, or other chemicals).

Authors: We agree with the reviewer that sensing IPTG with a biosensor might here not be useful *per se*, but as discussed in our manuscript, our work represents a proof-of-principle where we established a novel pipeline (*i.e.* feces extraction methods, plasmid replicons, dual plasmid system for inducible promoters) to show for the first time that native symbionts of the honey bee could be engineered and used as biosensors (see **lines 407-410**). For this reason, we submitted our manuscript for publication specifically as a "Methods and Resources" contribution to PLoS Biology. We would also like to stress that we are working with non-model microbes for which very little genetic tools were available. We understand that our work may appear simple if one considers the widely used chassis *Escherichia coli*, however, we believe our achievements are far from trivial in light of how poorly genetically tractable these bacterial species are.

We are currently developing other sensors in *S. alvi* to monitor more biologically relevant signals and learn more about the honey bee gut environment, but they will be part of a distinct manuscript. However, we have now tested the generalization of our sensor in different classes of Proteobacteria found in the honey bee gut, namely *Gilliamella apicola* and *Bartonella apis*, as well as in an additional strain of *S. alvi*. They are not closely related species, and it is evident that most genetic tools, including plasmid replicons, will not work universally in all these species, as exemplified with our work comparing replicons in *B. apis* and *S. alvi* (see Table 1 of our manuscript). For this, we used conjugation to transfer the pAC17V3, pAC17V5 and pAC17V4 plasmids (Supplementary Figure 6) in different strains for each species.

Plasmids from the pAC17V4 and pAC17V5 systems were not stably maintained over time and/or our genetic circuits were mutated in the strains tested. We believe that this is because of replicon incompatibilities and/or metabolic burden/toxicity. However, during the limited time given for this revision, we did not have time to systematically test replicon compatibilities in all the strains. However, the simpler circuit pAC17V3 (Supplementary Figure 6) was stably maintained. This allowed the two strains *Gilliamella apis* ESL0169 and *Gilliamella apicola* wkB7, as well as the strain *S. alvi* ESL0693 isolated from stingless bees, to respond to IPTG (see **new data below**; added to our manuscript as a **new Supplementary Figure 7** in the result section **lines 266-272: "***When transformed together in S. alvi, the plasmids enabled cells to respond in vitro to IPTG with a 7-fold increase in GFP expression (Fig. 5b).* ***Additionally, we wondered if other species of the honey bee gut microbiota could also be engineered to respond to IPTG. We tested our three genetically stable constructs in the two γ-Proteobacteria Gilliamella apicola wkB7 and Gilliamella apis ESL0169 from the honey bee, as well as in another strain of S. alvi isolated from stingless bees (Supplementary Fig. 7). We showed that all three bacteria engineered with our one-plasmid system pAC17V3 (Supplementary Fig. 6a) were able to respond to the presence of IPTG in vitro (Supplementary Fig. 7)***")*.

We therefore not only demonstrated that native symbionts other than *S. alvi* wkB2^T could be engineered with our circuits to sense IPTG, but we also showed that

symbionts of other bee species could be reprogrammed similarly. The fact that the dual plasmid system pAC17V5 could not be used with those *Gilliamella* and *Snodgrassella* strains is a testimony to the difficulty of genetically engineering these environmental undomesticated symbionts. We think the system could be functional in other symbionts, but this would require extensive work to first characterize all the replicons in each species (similarly to what we did for *S. alvi* and *B. apis*, shown in Table 1, Fig. 3 and Supplementary Fig. 2) before amending the IPTG-inducible circuit accordingly.

We have now added this point in our discussion (see lines **407-428**: "*Lastly, we designed a genetically stable inducible system based on two compatible replicons. As a proof of concept, we utilized it to reprogram S. alvi to sense IPTG in situ, therefore providing the first evidence that a prevalent honey bee symbiont can be engineered to behave as a biosensor (Fig. 5).* ***We also tested if the set of IPTG-inducible circuits we developed could be used to reprogram other symbionts, including strains from the genus Gilliamella, as well as another strain of S. alvi isolated from the microbiota of stingless bees (Supplementary Fig. 7). While the dual-plasmid system pAC17V5 was unfortunately not stably maintained in these species with its current design, most likely because of replicon incompatibilities, the RSF1010-based plasmid pAC17V3 allowed cells to respond to IPTG in vitro. These data are promising for the generalization of symbiont engineering as biosensors for the honey bee gut environment. However, as the gut microbiota is comprised of distinct and not closely related species, it is evident that most genetic tools (including plasmid replicons) will not work universally in all its members. This is a testimony to the difficulty of genetically engineering these environmental undomesticated symbionts(56), and more work will be required for the generalization of biosensor development across gut bacterial species. More importantly,*** *future research efforts should focus on the development of biosensors dedicated to more biologically relevant chemical signals, such as oxygen, pH, pesticides, or short-chain fatty acids for instance.*").

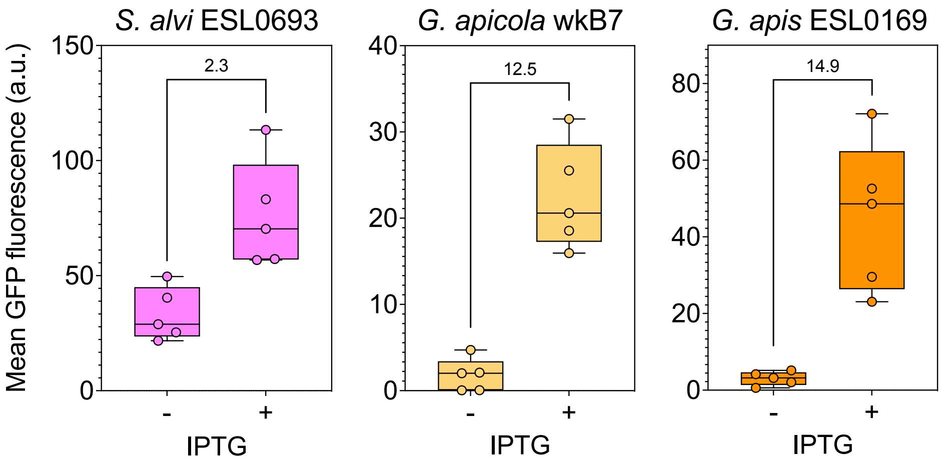

New Supplementary Figure 7. The IPTG-inducible construct pAC17V3 allows honey bee and stingless bee gut symbionts to sense and respond to IPTG. *Snodgrassella alvi* ESL0693, *Gilliamella apicola* wkB7 and *Gilliamella apis* ESL0169 cells engineered with the inducible

plasmid pAC17V3 respond to IPTG exposure *in vitro*. Graphs show box plots representing median value of GFP fluorescence of five biological replicates for each condition. Each replicate value is based on the fluorescence of cells grown as bacterial lawns onto solid media (TSA) supplemented with (+) or without (-) 1 mM IPTG. Fold-changes of average fluorescence between uninduced and induced cells are indicated. Fluorescence was determined from images taken by EPI fluorescence with a Fusion FX (Vilber Lourmat) apparatus (16 aperture, F-740 filter) and identical exposure times between conditions. Analysis was performed with the software Fiji (ImageJ2, version 2.9.0). The intensity measurement used was the mean intensity per unit area of bacterial lawn with the cell and media autofluorescence subtracted. Autofluorescence values were obtained for each strain based on the intensity measured from bacterial lawns of the corresponding wild-type cells (*i.e.* not bearing the pAC17V3 plasmid).

3) qPCR data reporting should follow generally accepted guidelines (i.e., MIQE). It does not look that MIQE has been followed.

   Authors: The MIQE guidelines were followed for qPCR data reporting and some clarifications on our method were added to the Materials and Methods section (see **lines 554-558**: "***Each reaction was performed in triplicate and no-template controls were added to every run to confirm that the reaction mixtures were not contaminated. The generation of specific PCR products by the qPCR primers was confirmed by melting curve analysis and*** *via observation of amplicons on an agarose gel (Supplementary Fig. 3).*").

4) Fig 2 can be a supplementary figure.

   Authors: We thank the reviewer for this suggestion, but we think this figure provides important information for the readers understanding of our work, including all subsequent figures 3, 4 and 5, and should thus remain as a main figure.

5) Fig 5. It looks that the sensor showed statistically significant sensing, but the fold change in response to different IPTG conditions (even at the highest concentration) is too small to be practical. Thus, along with my comments above, my enthusiasm for this work reduced, and this issue should be addressed.

   Authors: We agree with the reviewer that the fold-change observed in response to the presence of IPTG is modest, but it is sufficient to detect a statistically significant difference. We believe this is convincing evidence of functionality for a proof-of-principle work. Further efforts will be carried out, however, for the optimization of the dynamic range of future biosensors for biologically relevant compounds. We have now raised this point in our discussion (see **lines 410-413**: "***The fold-change induction observed in our system was modest, however, and might not be practical for easy assessment of the target compound concentration. Further optimization of the dynamic range should therefore be undertaken for future biosensors of biologically relevant molecules***").

<u>Reviewer #3</u>: This paper by Chhun et al. reports the development of molecular toolkits to genetically engineer the honey bee symbiont, Snodgrassella alvi, as a biosensor, using IPTG as a proof of concept. There is a need of using native symbionts for more accurate insights into the biology of host-associated tissues, since the majority of the reported biosensors rely on E. coli or a few other bacteria that would not readily persist in many non-model animal systems, including the honey bee. The current genetic toolkits which have been effectively used in the honey bee gut microbiome system are relatively large and complex, making them difficult to clone; furthermore, the lack of other replicons makes it difficult to build and use multiple constructs together. The authors results include a novel plasmid construct, pAC17V5, which is a circuit system composed of 2 plasmids, that allow S. alvi to exhibit a response to increasing doses of IPTG by reporting it as increasing GFP intensities.

The authors also propose a method for sampling and reporting bee gut contents via their faecal matter without the need to dissect the bees. The lack of established methodologies for faecal analysis in honeybees is a known problem, as honey bees do not typically defecate in laboratory conditions.
Overall, the paper provides a promising approach to address the challenges associated with studying host-microbiota interactions in the context of honey bee gut microbiota, and a strong proof-of-concept as to using engineered microbes as biosensors in this environment. It is slightly disappointing that they did not extend their work towards the sensing of natural compounds or environmental conditions that occur in the gut. Doing so may have revealed some novel biology and would have been a valuable addition to the study. As it stands, this study is nonetheless an impressive paper focused on method development in the bee gut system. The tools described here will likely be used by many researchers in the future.

While the paper is generally well written and the results are exciting, there are certain areas that are lacking in detail or require clarification.

Authors: We thank reviewer 3 for their very helpful suggestions/comments. We carefully took into consideration each of them and we have corrected our manuscript accordingly, when possible.

Line 98: How the bees were stunned should be mentioned here (CO2), in addition to in the methods.

Authors: We have now amended our text to include the method of stunning (see lines **98-99**: "*By applying a gentle pressure onto the abdomen of* ***CO_2-stunned*** *bees, a substantial amount of fecal matter could be retrieved*").

Line 99: In figure 1b, the average is given as 4.35 ul. Looking at the supplementary data, it seems that the 4.8 ul value mentioned in the text here excludes samples with 0 ul values. The authors should be consistent and include the zero value samples in their statistical calculations, since they represent real measured replicates.

Authors: We thank the reviewer for noting this inconsistency that is due to an oversight from our part. We have now included the zero values in our calculation and reported the revised value in our text (see line **100**: "*The volume of feces averaged* ***4.4 ± 2.4 µl*** *per bee (n = 18)*".

Figure 1d: How many times was this experiment replicated? Survival assays can suffer from high variance, especially in animal trials, and it would be better if these experiments were performed in triplicate.

Authors: The data reported in our manuscript result from bees housed in different cages that we considered as replicates. Given the seasonality of bee work, the revision period for this manuscript did not allow us to repeat the experiment another time. However, most of the results in this manuscript are based on feces collection that we performed up to 14 days post adult emergence (*e.g.* see Fig. 4) without noticing earlier death of the bees studied, thus being consistent with the presented survival data here.

Line 119: Is there a correlation between the amount of feces extracted from an individual and their survival probability?

Authors: Unfortunately, we did not record all the necessary data to investigate this and we therefore do not know.

Table 1: Please clearly define the criteria for a plasmid being considered "functional".

Authors: We replaced the term "functional" in our table to clarify that we considered plasmids to be functional when stably maintained within bacterial population (see **amended Table 1**).

Lines 175-181: This hypothesis can likely be tested by plating of their experiment of panel 4b on selective (spectinomycin?) media in addition to nonselective media. This would allow visualization of differences between the absolute CFU counts of Ab+ and Ab- treatments, and can confirm plasmid loss during culture.

Authors: We already provided what is suggested by the reviewer in our Figure 4, panel d. Indeed, the bacterial load values reported result from fecal samples plated onto selective (spectinomycin) media, as indicated lines **213-217** ("*Feces were sampled from these colonized bees and analyzed together with those collected for the measurement of plasmid stability. Fecal samples from the three treatment groups (i.e. +Ab, -Ab, +Gc) were ultimately examined by isolating engineered S. alvi onto selective solid media, allowing us to determine the levels of gut colonization through time (**Fig. 4d**).*"), as well as in the caption of figure 4. We amended the text to now highlight that our hypothesis is supported by the data (see lines **220-223**: "*This further suggests that **low plasmid maintenance values for the '+Ab' condition in Fig. 4a are indeed due to an artifact of the experimental setup, and that** maintaining the pVS1 origin of replication might be detrimental to cells' fitness and that stable colonization of the gut requires the addition of selection pressure.*").

Figure 4, caption title: Remove "in the honey bee gut.", since panel 4A is data from in vitro work.

Authors: We amended the caption title of our Figure 4 according to the reviewer's feedback.

Figure 4: It is not clear which plasmid versions are being tested here. In Fig. 2, there are plasmid variants constructed with combinations of GFP, Crimson, and ampR and specR. It

seems from the text that Crimson-specR was used, but it should be made explicitly clear, and the rationale given for this choice versus the other possible combinations.

Authors: The legend of the Figure 4 already stipulates at the end which plasmids were used (see legend Figure 4: "*Plasmids used for all panels in* S. alvi *were pAC10, pAC04, pBTK570 and pAC11, carrying the pVS1, pBBR1, RSF1010 and pTF-FC2 origins of replication, respectively.*"). We now added a clarification in our manuscript explaining the rational for choosing the crimson-specR plasmids (see **lines 164-166**: "***For this, we chose to test the set of vectors providing spectinomycin resistance (crimson/specR), as they prevent the growth of non-resistant satellite colonies typically obtained using ampicillin selection upon CFU estimation***.").

Figure 4d: For clarity, I suggest adding "-Ab" to the column with "+Gc", since I presume it is also without antibiotics.

Authors: We thank the reviewer for their suggestion. We preferred to keep the labels as they currently are, as we think it will decrease clarity and readability of our figure/legends if we would write '+Gc -Ab' for one condition. Instead, we amended the figure legend to make the point clearer (see legend Figure 4d: "*Concentrations of our different S. alvi strains isolated from the feces of bees co-colonized with the natural gut community* ***and that were fed sugar water without spectinomycin*** *(+Gc) was also analyzed.*").

Figure 4d: These samples were plated on selective media. There should have been controls to show that the +Gc microbiota do not have native resistance to spectinomycin at the concentrations used, which could confound the results. Even though the results for pVS1 suggest that the +Gc microbes are not resistant to their antibiotic, it is possible if each trial was done with a separate batch of gut inocula, there would be differences between the batches in terms of resistance.

Authors: We are confident that colony counts in the +Gc condition do not include unexpected growth of native gut bacteria as confounding factor. Indeed, we did perform controls in preliminary experiments where +Gc samples, as well as gut homogenates from nurse bees taken from different hives were plated onto selective media. We observed no growth during these experiments. In addition to the antibiotic selection, the media used (TSA) as well as the incubation conditions (microaerophilic) are themselves a selection pressure as most of the gut microbiota of honey bees would not grow in these specific conditions because of specific nutrients and/or anaerobic growth requirements. Lastly, as pointed out by the reviewer, the results for pVS1 show that no native bacterium from the +Gc inoculum is resistant to spectinomycin, and we used the same batch of gut homogenate across the different conditions.

Figure 4d: There are many zero values for the CFU counts, which require explanation. The median values for each treatment listed in the supplementary raw data file do not correspond to the indicated medians in the figure, nor do the interquartile ranges seem to make sense. It seems the authors have excluded the zero values from their analysis, and this should be clearly stated and justified.

Authors: We have indeed excluded the zero values from the reported median, as they are not representative of the bacterial load present in the gut. Instead, we used those values to estimate the number of non-colonized bees. We believe that reporting the actual bacterial

load of colonized bees, alongside the rate of those colonized bees is more nuanced and informative. We amended the figure legend to make the point clearer (see Figure 4d legend: *"**Bees for which we did not detect engineered bacteria were considered non-colonized individuals.** The rate of colonized bees (r) and the number of bees sampled (n) are provided. Corresponding median values of colonized bees **only** are represented by colored horizontal bars with interquartile ranges."*).

Figure 4: Analyses 4c and 4d seem to have been performed on the same samples. Therefore, it is odd that the number of samples (n's) analyzed in the panels are different. This should be explained. For instance, were the samples chosen for plating on non-selective media (Fig. 4c) a randomly chosen subset from 4d?

Authors: The plasmid maintenance values (Fig. 4c) were obtained from fecal samples spotted onto non-selective media, so we could compare the number of fluorescent CFUs over the overall number of CFUs, as explained in our material and methods section (see lines **614-623**). These values could thus only be estimated from bees with *S. alvi* in their gut (whether engineered bacteria still carrying our plasmid or those who have lost the plasmid). Samples used for our analysis shown in Fig. 4c are therefore not a randomly chosen subset from Fig. 4d but the subset corresponding to colonized individuals for which it was possible for us to determine plasmid maintenance levels. We amended our figure legend to make this point clearer (see Figure 4c legend: "***c** from feces (in vivo) collected 7 (orange) and 14 (red) days after gut mono-colonization of bees continuously fed sugar water supplemented with (+Ab) or without (-Ab) spectinomycin. **Only samples for which we detected bacterial colonies upon plating the fecal material were considered.***").

Figure 4: pBBR1 panel 4c -Ab treatment shows a drastic drop from day 7 to day 14; however, there are still many cells (around 10^3) from the day 14 sample as shown in the corresponding panel 4d -Ab treatment day 14. Do the authors imply that the lack of fluorescence is from the loss of the plasmid after growth on non-selective media, as in the case of pVS1? Also, it is not clear whether the zero values for plasmid maintenance in panel 4c is because they did not observe growth, or whether the colonies did not fluoresce. There should be mention of how many colonies were counted to obtain the percentages shown.

Authors: The plasmid maintenance values are expressed as percentages. As such, it is possible to obtain very low plasmid maintenance levels while still having a relatively 'large' number of cells. However, 10^3 is about three orders of magnitude less than the maximum bacterial load detected in our experiments and we therefore do not consider this a lot of bacteria. As explained above, the zero values for plasmid maintenance correspond to colonies that did not fluoresce, as we only considered colonized bees (*i.e.* individuals for which we observed bacterial growth upon plating their fecal material). We now mention in our Materials and Methods section the median value of colonies counted for the plasmid maintenance values (see **lines 622-623**: "***A median value of 69.5 colonies were counted and analyzed to obtain the plasmid maintenance percentage for each sample***").

Lines 196-198: The assertion that pBBR-1 is lost quickly contrasts with line 220, where they say that this plasmid is stably maintained even without antibiotics.

Authors: In line 220 (now 223), we do not claim that the plasmid with a pBBR-1 replicon is stably maintained without antibiotics, but that cells carrying it can "establish persisting

communities", which is a distinct point. As mentioned above, a low plasmid maintenance level can still represent detectable number of cells still bearing the plasmid, although a significant portion of the population loses it. We also clarify this point later in the text when we discuss that (lines **226-234**) "*Bacterial concentrations and success rates of colonization found for each strain were also in line with the varying degrees of plasmid maintenance established in this study. […] while the less frequently maintained vector pBBR1 resulted in fecal bacterial populations eighteen times smaller. These observations were also consistent with the rates of non-antibiotic-treated honey bees successfully colonized by each strain, as on average 50%, 61% and 76% of bees were colonized by S. alvi transformed with the pBBR1, RSF1010 and pTF-FC2 replicons, respectively.*".

Line 185: Please specify what is the CFU of the initial inoculum was, since this would help you interpret whether the bacteria are actively colonizing vs. persisting/dying over time.

Authors: We have now specified in our material and method section the average number of CFU fed to the bees (see lines **590-593**: "*Gut mono-colonization of the microbiota-depleted bees was carried out by individually feeding them 5 μl of solutions of S. alvi cells at an OD_{600} of 0.1 resuspended in 1:1 (v/v) 1X PBS:sucrose water, **representing an average of 1.9 $10^4 \pm$ 1.89 10^3 bacteria per inoculum.***") and added a clarification on our methods to check that our microbiota-depleted bees were not contaminated prior to colonization (see **lines 584-589**).

Line 214: Specify what is the "selective solid media". Spectinomycin?

Authors: The selective solid media is indeed supplemented with spectinomycin, as specified in our material and methods (see lines **616-618**: "*Diluted feces were then plated on TSA solid media supplemented with or without 60 μg ml^{-1} spectinomycin, as appropriate.*"). To make it clearer, we also amended the legend of Figure 4 to include the spectinomycin supplementation ("*Those CFUs values were obtained by plating diluted feces onto selective media **(i.e. supplemented with spectinomycin)**.*".

Line 250: Please provide more details as to the experiment where it was determined the pBTK552 plasmid had low stability. For instance, what was the time interval over which stability was tested?

Authors: We amended our Supplementary Figure 5 legend to include the duration of the experiment ("*Each replicate value is based on the average fluorescence of at least 9,000 S. alvi cells measured by flow cytometry, which were grown in **3 ml of TSB** with (+) or without (-) IPTG **for 3 days**.*").

Figure 5e and text Lines 604-609: It is unclear here exactly which types of images were taken for fluorescence quantification. Is the image in panel 5d an representative image? How big of an area was imaged (can you provide dimensions)? How big were the areas of host and bacterial cells selected for analysis? How many of each type of cell is expected to be included in such a selection?

Authors: The image in panel 5d is a representative image that was taken at low magnification (10X objective), as it showed better the colonization localization. The images used for GFP quantification were simply taken at higher magnifications (63X oil objective),

which allowed for easier selection of precise areas containing bacterial cells, with dimensions 134.6873 μm (512 pixels) in width and height, with a depth of 1.5200-1.8400 μm. We amended the material and methods section to include these information (see lines **641-643**: "*Images were acquired with a 512x512 pixel format size* ***(134.6873x134.6873 μm and depth of 1.5200-1.8400 μm)****, an averaging of 4 images and a 63X oil objective*"). The size of areas from which we quantified gfp varied between pictures as we used the freeform tool to enclose colonized areas, but the gfp intensities reported were normalized to unit area. We did not select areas for quantification that contained host cells, but only areas where *S. alvi* biofilm was found within gut crypt. Clarification on the selected area for analysis and cell type has been added to the text (see lines **646-648**: "*In Fiji, each image was split into DAPI and GFP channels and the freeform tool was used to draw three densely colonized areas of each ileum section, based on the DAPI channel.* ***Host cells were excluded from the area of interest***.").

Lines 466-482: A new electroporation method was developed, which is mentioned as a faster and simpler method to transform S. alvi. This is compared to the previous conjugation method. However, there are no data provided as to the transformation efficiency of this new electroporation method.

Authors: The number of colonies obtained after electroporation varies greatly based upon the plasmid transformed and the batch of competent cells prepared but we typically could obtain between 1 to > 2,000 colony after electroporation, corresponding to an average transformation efficiency of 6.6 $10^3 \pm 1.92\ 10^3$ CFU μg^{-1} of DNA. We have added this information to the corresponding material and methods section (see lines **511-512**:" *Colonies resulting from successfully transformed cells typically appeared after 3-4 days****, and transformation efficiencies averaged 6.6*** $10^3 \pm 1.92\ 10^3$ ***CFU*** μg^{-1} ***of DNA***.").
